# Gastric perforation without generalized peritonitis; A very rare complication after necrosectomy for necrotizing pancreatitis

**DOI:** 10.12669/pjms.323.9726

**Published:** 2016

**Authors:** Kamran Hakeem Khan, Mohammad Farid Khan, Tariq Jabbar Khan

**Affiliations:** 1 Dr. Kamran Hakeem Khan, Registrar Department of Surgery, North West General Hospital & Research Center, North West General Hospital & Research Center, Hayatabad, Peshawar, Pakistan; 2 Dr. Mohammad Farid Khan, FRCS. Consultant Department of General surgery, North West General Hospital & Research Center, North West General Hospital & Research Center, Hayatabad, Peshawar, Pakistan; 3 Dr. Tariq Jabbar Khan, FRCS. Consultant Department of General surgery, North West General Hospital & Research Center, Hayatabad, Peshawar, Pakistan

**Keywords:** Peritonitis, Necrosectomy, Necrotizing Pancreatitis

## Abstract

Gastric perforation is a very rare complication of necrotizing pancreatitis. We present an interesting case of gastric perforation after necrosectomy for necrotizing pancreatitis without generalized peritonitis. Abdominal drain was seen inside the stomach on endoscopy and there were no clinical features of generalized peritonitis even after 10 days of surgery. Patient was re-explored. The drain was removed and stomach was primarily repaired. The patient recovered uneventfully and was discharged home on 6^th^ post operative day. On follow-up visit after one month patient was doing very well and had no complications.

## INTRODUCTION

While the debate and controversies on treatment options for the optimal management of severe necrotizing pancreatitis (SNP) are still ongoing, open surgical debridement remains the “gold standard” for the treatment of infected pancreatitis and pancreatic necrosis. Necrosectomy and subsequent closed continuous lavage of the lesser sac is, among the open necrosectomy techniques, the one with the lowest morbidity.^1^ It is generally agreed that treatment of severe necrotizing pancreatitis should be conservative in its early phase, aiming at fluid replacement, sufficient analgesia and prevention of organ failure.^2^-^5^ Surgery might be considered as an option for the diseaseat a later phase. In fact, surgical debridement is still considered the treatment of choice in patients with proven infected pancreatic necrosis, given that they present with organ failure.^6^ The use of minimally invasive techniques in combination with conservative therapy can decrease treatment time, the rate of morbidity and mortality in patients with acute necrotizing pancreatitis.^7^

Along many other complications of necrosectomy, a very rare on is gastric perforation. This case was unique because the gastric perforation after necrosectomy was without symptoms of generalized peritonitis. The perforation was well plugged around the drain in stomach and hence generalized peritonitis was avoided in the presence of gastric perforation.

## CASE REPORT

A 56 years old lady from Afghanistan presented in the surgical outpatient department of our hospital with a history of laprotomy and necrosectomy for necrotizing pancreatitis in Afghanistan 10 days back. Her chief complaints were epigastric pain since surgery and fluid draining through the abdominal drain soon after drinking any liquid. This drain was on the left side of the upper abdomen. The color and consistency of fluid in drain was very similar to the fluid she would drink. On clinical examination she had no signs of septicemia and generalized peritonitis. She was afebrile with a regular pulse of 84 per minute and blood pressure of 115/75mmHg. The laprotomy wound dressing was dry and clean. There were two abdominal drains on either side of abdomen. She was admitted in ward and was investigated. Blood tests revealed WBC=1210 and shift to the left, rest of the blood count, serum electrolytes, liver function tests and renal function tests were in normal limit. Her hepatitis serology for HBS and HCV was negative. Her gastrograffin study of upper gastrointestinal tract showed external drainage of the contrast from the stomach through a drainage tube in her left upper abdominal area with suspicion of malposition of drainage tube with its tip in the stomach. CT scan showed abdominal collections in lesser sac and peri pancreatic region approximately about 60ml and 65ml respectively. Drainage tube was noticed entering through the left hemi abdominal wall entering the greater curvature and ending up in the gastric lumen. Upper gastrointestinal endoscopy revealed multiple small gastric ulcers and tip of the abdominal drain inside the stomach along the greater curvature. Patient was re-explored. Per operative findings were:

**Fig.1:**
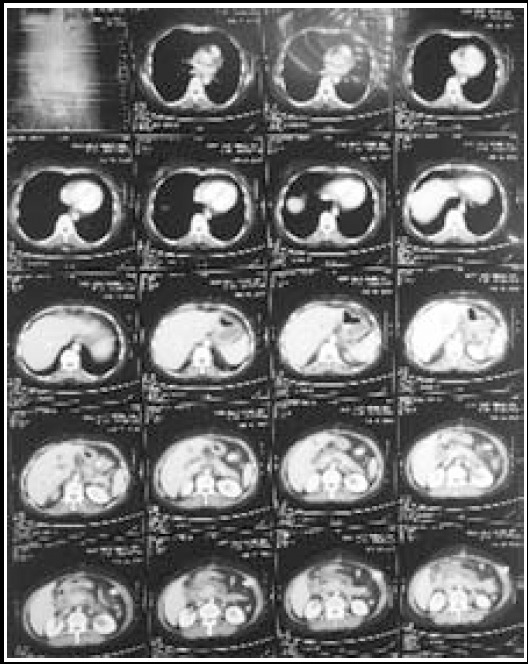
CT scan film.

1. Left sided abdominal drain was seen, piercing the greater curvature of the stomach. About 2cm, from the tip of the drain, was found inside the stomach.
2. Omental mass in the area of the pancreatic bed, adherent to loops of small bowel.
3. Rest of the bowel was normal and no other abnormality was seen.

The abdominal drain from the stomach was removed. The edges of the gastric perforation were refreshed. Primary repair of the gastric perforation with omentopexy was done. Oral fluids were commenced on 4^th^ post operative day. Her post operative recovery was uneventful. She was discharged home on 6^th^ post operative day. Her first follow up was planned two weeks after the surgery. She had no active complaints and was taking normal diet, tolerating well and had normal bowel habits.

**Fig.2:**
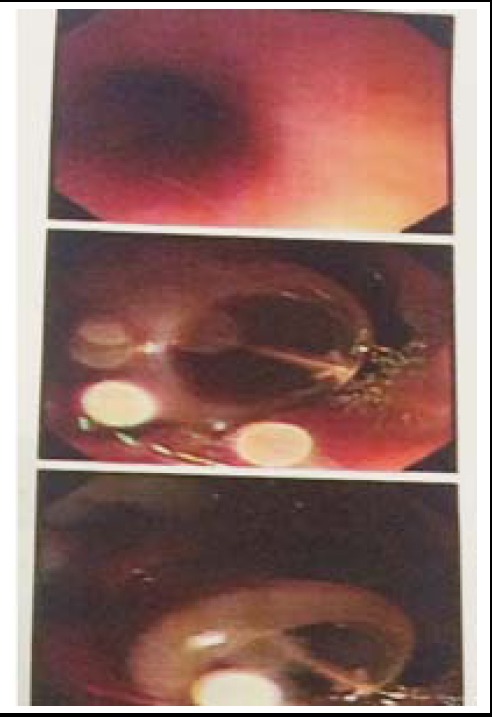
Endoscopy picture.

**Fig.3:**
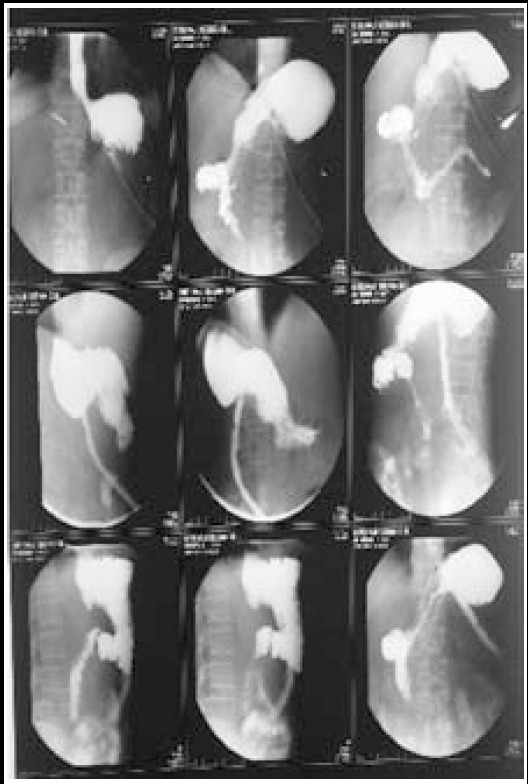
Gastrograffin film.

## DISCUSSION

Acute pancreatitis is still associated with significant morbidity and mortality. Current management guidelines are sometimes equivocal, particularly in relation to the surgical treatment of severe disease. Despite a relative shortage of prospective randomized trials, there has been a significant change in the surgical management of acute pancreatitis over the past 20 years. This change has been away from early aggressive surgical intervention towards more conservative management, except when infected necrosis is confirmed. A formalized approach with appropriate use of the various non-surgical and surgical options is feasible in the management of severe acute apancreatitis.^8^

In a study of 88 patients with necrotizing pancreatitis Connor S et al found that almost all patients undergoing necrosectomy developed significant early or late complications or both. Multi-organ failure and post operative hemorrhage were independent predictors of mortality.^9^ To our knowledge there are no reports of gastric perforation in patients with necrotizing pancreatitis without clinical features of generalized peritonitis. The literature shows cases with colonic perforation and large bowel obstruction after necrotizing pancreatitis^10^ but they all presented with peritonitis. Lin CK et al in their study found that acute gastrointestinal mucosal lesions in patients with acute pancreatitis can range from simple gastritis and erosions to ulceration or bleeding. The majority of ulcers are located in the stomach and duodenum and to a very lesser extent in the esophagus.^11^ Chen TA et al found that 65% of patients of acute pancreatitis complicate with acute gastrointestinal mucosal lesions and this percentage is more in necrotizing pancreatitis.^12^ Necrosectomy and closed lavage for necrotizing pancreatitis carries an overall mortality of 25-50%^13^ and peritonitis further increases the rate. A case reported by university hospital of Tubingen, Germany, describes endoscopic treatment of gastric perforation caused by acute necrotizing pancreatitis using over-the-scope clips. In our patient, laprotomy was performed by consultant surgeon, drain was removed and gastric perforation repaired in traditional two layers.

Another important factor is that placement of abdominal drain plays a vital role in reducing the chances of peritonitis in our patient and recognition of perforation as well. This not only shows the importance of traditional placement of abdominal drain after necrosectomy but contradict a study performed by Kevin C Conlon et al, which showed that surgically placed intra peritoneal drains reduces the rate of either death or complications associated with pancreatic resections.^14^
